# SARS-CoV-2 Disease Adjuvant Therapies and Supplements Breakthrough for the Infection Prevention

**DOI:** 10.3390/microorganisms9030525

**Published:** 2021-03-04

**Authors:** Alessio Danilo Inchingolo, Angelo Michele Inchingolo, Ioana Roxana Bordea, Giuseppina Malcangi, Edit Xhajanka, Antonio Scarano, Felice Lorusso, Marco Farronato, Gianluca Martino Tartaglia, Ciro Gargiulo Isacco, Grazia Marinelli, Maria Teresa D’Oria, Denisa Hazballa, Luigi Santacroce, Andrea Ballini, Maria Contaldo, Francesco Inchingolo, Gianna Dipalma

**Affiliations:** 1 Department of Interdisciplinary Medicine, University of Medicine Aldo Moro, 70124 Bari, Italy; ad.inchingolo@libero.it (A.D.I.); angeloinchingolo@gmail.com (A.M.I.); drciroisacco@gmail.com (C.G.I.); graziamarinelli@live.it (G.M.); mtdoria51@gmail.com (M.T.D.); denisahazballa@gmail.com (D.H.); luigi.santacroce@uniba.it (L.S.); francesco.inchingolo@uniba.it (F.I.); giannadipalma@tiscali.it (G.D.); 2 Department of Oral Rehabilitation, Faculty of Dentistry, Iuliu Hațieganu University of Medicine and Pharmacy, 400012 Cluj-Napoca, Romania; 3 Dental Prosthesis Department, Medical University of Tirana, UMT, Rruga e Dibrës, Tirana 1001, Albania; editxhajanka@yahoo.com; 4 Department of Innovative Technologies in Medicine and Dentistry, University of Chieti-Pescara, 66100 Chieti, Italy; ascarano@unich.it; 5 Department of Biomedical, Surgical and Dental Sciences, School of Dentistry, University of Milan, UOC Maxillo-Facial Surgery and Dentistry, Fondazione IRCCS Ca Granda, Ospedale Maggiore Policlinico, 20100 Milan, Italy; marco.farronato@unimi.it (M.F.); gianluca.tartaglia@unimi.it (G.M.T.); 6 Human Stem Cells Research Center HSC of Ho Chi Minh, Ho Chi Minh 70000, Vietnam; 7 Embryology and Regenerative Medicine and Immunology, Pham Chau Trinh University of Medicine Hoi An, Hoi An 70000, Vietnam; 8 Department of Medical and Biological Sciences, Via delle Scienze, Università degli Studi di Udine, 206, 33100 Udine, Italy; 9 Kongresi Elbasanit, Rruga: Aqif Pasha, 3001 Elbasan, Albania; 10 Department of Biosciences, Biotechnologies and Biopharmaceutics, Campus Universitario, University of Bari, 70125 Bari, Italy; andrea.ballini@uniba.it; 11 Department of Precision Medicine, University of Campania, 80138 Naples, Italy; 12 Multidisciplinary Department of Medical-Surgical and Dental Specialties, University of Campania Luigi Vanvitelli, Via Luigi de Crecchio, 6, 80138 Naples, Italy; maria.contaldo@unicampania.it

**Keywords:** SARS-CoV-2, COVID-19, pandemics, resveratrol, oral mucosa, furin, cytokine storm syndrome, microbiome, vaccines, endovir stop spray

## Abstract

The SARS-CoV-2 (severe acute respiratory syndrome coronavirus 2) is a high-risk viral agent involved in the recent pandemic stated worldwide by the World Health Organization. The infection is correlated to a severe systemic and respiratory disease in many cases, which is clinically treated with a multi-drug pharmacological approach. The purpose of this investigation was to evaluate through a literature overview the effect of adjuvant therapies and supplements for the SARS-CoV-2 infection. The research has analyzed the advantage of the EK1C4, by also assessing the studies on the resveratrol, vitamin D, and melatonin as adjuvant supplements for long hauler patients’ prognosis. The evaluated substances reported important benefits for the improvement of the immune system and as a potential inhibitor molecules against SARS-CoV-2, highlighting the use of sartans as therapy. The adjuvant supplements seem to create an advantage for the healing of the long hauler patients affected by chronic symptoms of constant chest and heart pain, intestinal disorders, headache, difficulty concentrating, memory loss, and tachycardia.

## 1. Introduction

### 1.1. Epidemiological and Demographic Characteristics

Among the most common clinical symptoms of COVID-19, fever, dyspnea, asthenia, cough, anosmia, and dysgeusia are listed as well as few gastrointestinal symptoms, headache, and sore throat, leading in the most severe cases to acute respiratory distress syndrome with bilateral interstitial acute pneumonia, multiple organ failure, and death [1,2,3,4,5,6,7]. A study showed that chilblains, urticaria, and tremors have been reported as associated symptoms of the patient with COVID-19 [8,9,10,11,12,13]. Some isolated cases were recorded; there were only three cases in Madrid (two suspected and one confirmed) of herpetic-like vesicular lesions in the oral cavity with pain, desquamative gingivitis, and ulcers [14,15]. The average age of 2019-nCov infected patients is 55.5 years, while for mortality (case fatality rates CFR), age is 75 years, and it gets higher in the 80s age group [16]. The number of deaths is higher in the elderly population with comorbidity, which enforces the key role that the immune system plays in the control of persistency of the SARS-CoV-2 virus [16]. It is noted that the decay of the immunity is observed in ageing and, therefore, the SARS-CoV-2 virus may get an easier access on the respiratory tract in elder patients. Men are more affected than women (67%), as there are more smokers in the male population and the female immune system has a better antibody system lined to the X chromosome [7,17,18,19,20,21,22,23,24,25,26,27]. The role of smoking has been initially hypothesized as risk factor for the COVID-19. Indeed, smokers and patients affected by chronic obstructive pulmonary disease (COPD) have a higher quantity of ACE2 receptors, which also are the receptors of access for the COVID-19 virus. Moreover, the ex-smokers highlighted a peculiar genic expression included in non-smokers and active smokers [19,20]. A report analyzed 28 studies and reported that the smokers were more susceptible to contract COVID-19 compared to the non-smokers-population [19,28]. Another study reported a percentage of 12.4% of smokers hospitalized for COVID-19 in an intensive care unit with invasive/non-invasive assisted mechanical ventilation, while a percentage of 4.7% of the non-smokers needed of the intensive therapy [19]. A higher predisposition to COVID-19 has been reported for male subjects (67%) compared to the female patients (288 million men vs. 12.6 million of women), while a predominant smoking habit is present in the occidental male population [19]. In another study that uses the sequencing of single cells, it has emerged that the expression of ACE2 was predominant in Asian men, which was significantly higher in the current smokers of Asian ethnicity than the non-Asian smokers [29]. Diseases such as diabetes (7.3%), chronical respiratory infections (6.3%), cardiovascular problems (10.5%), hypertension (6%), and tumors (5.6%) are comorbidities constituting a high risk of infection [21,30,31]. The early diagnosis together with adequate prevention methods (social distancing, use of personal protective equipment, such as face masks and wash handing with alcohol solutions) are important to contain and contrast the 2019-nCoV spreading [10,32]. After several studies, the WHO has confirmed that the diffusion of the 2019-nCoV mainly occurs through saliva droplets [33,34,35,36,37,38] and nasal secretions and tears, and in a lower measure through feces, urine, sperm and blood. Therefore, the oral cavity is the main access and exit. In the assessment of the contagion of saliva, it is important to consider the “*time of physical decay*” depending on the droplets size (Flügge’s droplets), the speed of emission (sneeze or cough), the moisture content in the room and the air exchange, and the “*biological decay*”, namely how long the virus keeps infecting in saliva droplets. The biological decay is caused by dehydration, ultraviolet rays, and chemical products [39,40,41]. There are some studies performed about the persistency of the 2019-nCoV in aerosol and different surfaces (plastic, steel, copper, carton) [24,42,43,44,45,46]. The stability of SARS-CoV-2 on different surfaces has been studied by infecting them in a room at 22 °C and with a moisture rate of about 65%. The results have shown that no traces have been reported after half an hour on printed paper and tissue paper and smooth surfaces such as wood and banknotes, and after seven days there were no traces on plastic and stainless steel. Instead, the data about the surgical face masks are interesting, where on the external surfaces, after seven days, there have been some important traces of active virus [39,47,48]. Some recent studies showed that SARS-CoV-2 remains active for up to nine days. The plastic and the stainless steel are the surfaces on which the SARS-CoV-2 virus lives longer. The biological decay depends on temperature (at 30–40 °C, virus expires), and at a temperature of less than 4 °C, SARS-CoV-2 may remain active for up to 28 days. Using for the disinfection of surfaces: 0.1% sodium hypochlorite or 62–71% ethanol coronavirus notably reduces its infecting action on the surfaces within 1 min of exposure [28,40,43,49,50,51]. Shanna Ratnesar-Shumate et al. showed some encouraging results about the capacity of the sun light to quickly disactivate the SARS-CoV-2 virus. Data show that the natural light has a higher disactivating power and may effectively disinfect nonporous contaminating materials [52]. Even the simulated sun light has inactivated the coronavirus SARS-CoV-2 on infected samples performed on stainless steel. The virus has been disactivated in 90% in just 6.8 min in a salivary solution, while in 14.3 min in the laboratory on lands of culture [52]. SARS-CoV-2 remained active in the air for the duration of the experiment, namely 3 h.

### 1.2. COVID-19 in Childrens

Some studies on the ongoing infections in babies based on some recent epidemiological data coming from Norway, Iceland, South Korea, and China, even if analyzed on different sized samples, all confirm the same infection rate, namely that babies represent 1–5% of the infected population, and most of them are asymptomatic or they show a slight or moderate symptomatology, higher in the male population [53,54,55,56,57,58,59,60,61]. The 90% of babies with severe evolution of the disease interests the age group from zero to two years [58]. A study performed on South Korea babies showed that the rate of severe cases has been 10.6% of babies aged less than one year, 7.3% in the group aged from one to five, 4.2% of babies aged from six to 10, 4.1% of those aged between 11 and 15, and 3.0% among people aged 16 or more [58]. Those numbers are uncertain, above all for younger children, in which a high rate (71%) has been diagnosed without a test and in 13% of cases without symptoms [58]. Lu et al. have issued a similar report; on 1391 subjects less than six years old, 171 (12.3%) have resulted positive. Only three of them needed intensive care; all three cases were already affected by severe diseases (hydronephrosis, leukemia, and intestinal invagination) [57]. Twenty-seven babies on 171 (15.8%) did not have symptoms or radiological signs of pneumonia [57,58,62]. The average age of the infection was of 6.7 years. The fever was always present in the 41.5% of babies. Frequent cough and pharyngeal erythema, increased heart rate, respiratory frequency, and gastrointestinal diseases. A total of 27 patients (15.8%) showed no clinical symptoms of infection or radiological signs of pneumonia. A total of 12 asymptomatic patients showed radiological signs of pneumonia, while the most common radiological evidence was the bilateral opacity of lungs (32.7%). In March 2020, the death of a 10-month-old baby affected by an intestinal invagination with multiorgan failure was reported; the subject died after four weeks from the hospitalization [59,63]. A number of 21 patients were in stable conditions in general departments, and 149 were released from the hospital. Skin lesions, similar to vesicles on hands and feet, are among the new clinical signs; it is supposed that they are related to the peripheral circulatory system, which may lead to necrosis areas [64,65,66]. The presumed lower occurrence of infection in babies may be linked to the structural and functional immaturity of the cellular receptor ACE2 site by offering less affinity to the virus spike [67]. Differently to the infected adults, most of the infected babies seems to be a milder clinical course. Frequent symptomatic infections are a sign that infected babies may be a silent element of infection [68]. This is an important consideration for prevention and containment measures. On March 31, seven deaths have been reported in the world [62]. More recent studies reported that SARS-CoV-2 manifests itself with a more favorable clinical prognosis in pediatric patients compared to the adult subjects. In fact, children have a lower mortality than adults, which is around 0.06% in the 0–15 age group [69]. Studies on the Italian population reported that the total confirmed pediatric cases were 1.8%, with an average age of 11 years and with a slight prevalence of males; of these, 13% were hospitalized, and 3.5% were hospitalized in intensive care. The risk increases inversely proportional to age and the presence of comorbidities and the patients that showed critical clinical evolution are 0.6% of the children, but 50% of them are less than one year old [69,70]. In symptomatic children, with/without non-severe clinical symptoms, SARS-CoV-2 remains longer in the upper respiratory tract and in the faeces, manifesting symptoms not very present in adults: increased secretions in the upper respiratory tract and phenomena of gastroenteritis that facilitate the spread of the virus through the respiratory and fecal–oral route [71,72,73]. The English variant SARS-CoV-2 B.1.1.7 seems to have had greater diffusion among children and adolescents, although the type of clinical course proved to be equally not critical as the initial strain. There were no age differences or differences in patients with comorbidities or percentages of black and Asian patients [74]. From data reported in the scientific literature, in the pediatric and adolescent population there appears to be a correlation between SARS-CoV-2 infection and the onset of a new rare syndrome, called multisystem acute inflammatory syndrome (MIS-C), which, presenting some clinical manifestations typical of Kawasaki disease, is also called “*Kawasaki syndrome*” [65]. Children are less affected than adults for various hypotheses. Some studies report a lower presence of the angiotensin converting enzyme 2 receptor (ACE2) and a difference in the distribution, maturation, and functioning of this viral receptor, and possibly a lower presence of ACE2 in children’s lungs [61]. Another possible hypothesis is the lower presence of endothelial damage related to age, cardiovascular disease, diabetes mellitus, smoking, and lack of vitamin D, all of which are considered risk factors for severe COVID-19. In fact, the presence of endothelial damage facilitates and increases the inflammatory response from SARS-CoV-2 that causes vasculitis and activates the coagulation pathways and the formation of microthrombi that cause serious thrombotic complications such as heart attacks and strokes [61]. In addition, children and adolescents do not have an aging immune system or immunosenescence, which reduces the ability of B cells to produce antibodies against new antigens and to recognize pathogens [61]. According to the literature concerning the therapies recommended in children infected with COVID-19, it was established that children and adolescents, having a benign clinical course, should choose the pharmacological treatment other than supportive therapy only in the most serious cases [75]

### 1.3. Dental Medical Care

During everyday dental activity, there is a strong chance that the aerosol material includes supra-and-subgingival virus, blood, and microorganisms [24,44,45,46,48]. At the moment, it is impossible to determine the exact infection risk represented by the aerosol material, but it is a real risk, and we need to eliminate or reduce it as much as possible during clinical procedures. The use of personal protective barriers such as face masks (surgical face masks, FFP2, P 100, FFP3), gloves and eye protection, single-use-gowns, visors, double-inlet, and premises sanitization, will eliminate a great part of danger included within droplets coming from the operating sites [27,44,75,76]. The aerosol or the droplets may be present in the air of the operating room after a procedure even for three hours [44]. This means that, after a dental procedure, if the operator removes a protecting barrier, such as a face mask, in order to talk to his patient, there is a potential contact with contaminated material in the air. Therefore, there is the need to keep on wearing the protection equipment [53]. Moreover, a contaminated substance on the air may penetrate the ventilation system and spread all over the premises [45]. Another chance may be the use of a high efficiency particulate air filter, or HEPA filter, as well as the use of UV rays chambers in the ventilation system. Even though those systems are very expensive, they seem to reduce air contamination. A UV system is nowadays prohibitive for most dental practices. The use of silver salts and ozone sanitizers may be performed only at the end of the day for the long time required by both systems. This is not feasible because of the great quantity of the daily dental visits [76]. It has been shown that dental practitioners are subjected to exposure at SARS CoV-2 virus while performing dental treatments. The virus can enter the organism through the airways, represented by the oral cavity and nose, and also through the eyes; this is why doctors have to wear protective equipment in order not to be infected with the virus [44]. A method to reduce the infective risk is characterized by a hydrogen peroxide solution administration and two parts of water, repeated more times during the treatment. Indeed, the hydrogen peroxide is naturally generated by oral bacteria and intervenes by regulating the balance of the oral microsystem. In epithelial cells, the hydrogen peroxide through the enzymatic activity of the superoxide dismutase is catalyzed in superoxide ion [76]. Through this oxidative stress, the toll receptors and NF-Kb are activated. The same mechanism is triggered, with the viral infections by playing an important role in host immune system. For this reason, the cleansing of the nasal and oral mucosa with hydrogen peroxide would improve the response of the host of the viral infection, by reducing the viral load and breaking the diffusion risk [76]. The concentration at 6% of H_2_O_2_ in oral hygiene is recommended in the British Nationally Formulary, while in otorhinolaryngology, it is generally used in viral infections for gargling or asa nasal spray solution at 3% [76,77,78]. The use of a chlorhexidine at 0.2% or a mouthwash containing essential oil has not shown high neutralizing capacity for the virus [79,80,81]. During dental procedures, the use of a rubber dam would reduce the virus spreading, and we also have to take into consideration the root canal disinfection. The only source of contamination in the air comes from the tooth under treatment [82,83,84]. Additionally, the contamination can be reduced by using laser. The major benefit of this technology is that it reduces the quantity of aerosols that is produced [85,86,87]. The guidelines recommended by World Health Organization indicate the use of specific protective equipment that is composed of masks that have to have at least 94% power of filtration for the air particles, the use of eye glasses, and costumes that have to be waterproof [17]. Scarano identified in his study that the use of a specific type of mask (N95) determines the modification of the temperature underneath and discomfort [27]. The use of water-cooled rotary instruments and also ultrasonic ones generate a big amount of aerosols [17]. In order to reduce the viral spread dentists are able to use lasers, hand instruments when performing root scaling, double surgical suctioners, and dental rubber dam [17]. In a study performed by Herrera, the authors indicated the use of a combination of mouth rinses in order to reduce the viral load [88]. The combination includes N-hexadecyl pyridinium chloride, chlorhexidine, citrox, and cetylpyridinium chloride; also it includes essential oil and beta cyclodectrin [40].

## 2. Viral Genomics and Receptors

**Figure 1:**
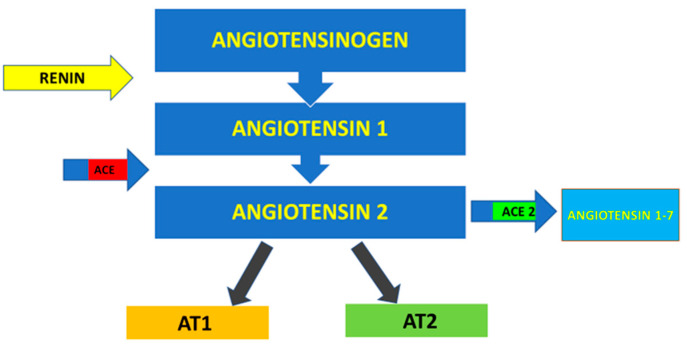
Homeostatic system of renin–angiotensin, figure designed by Giovanna Dipalma.

In nature, there is a great amount of virus DNA and RNA, both double (dsDNA or dsRNA) and single (ssDNA or ssRNA) filament. Single filament viruses also have the “*polarity*” (corresponding to the coding mechanism), positive or negative, or the ssDNA-, ssRNA-, ssDNA+, or ssRNA+ (e.g., coronavirus). The specificity of the genome modulates the replication of the cell (cytoplasmic or nuclear) [89]. When a copy of the genome of the virus enters one of the host cells, in a few hours, thousands of viral particles are formed, which are released in the external environment. In the RNA virus, there are no polymerase RNA; in the correction system of the transcription error, we may say that the replication of the virus easily occurs with errors. Therefore, those two elements (high number and error frequency) may explain the quick development of the virus and the continuous appearing of therapy-resistant-mutants, which are able to evade the attack to the immune system, continuously changing in response to adapt to the constant change of the genome [12]. Classification changes according to their nature, structure, genome, and type of reproduction. One of the features of the virus is the attack on the host cell, having receptors with related connections on its surface. When the connection with those related receptors occurs, the virus enters and invades the host cell by transferring its own genome inside, DNA or RNA. Immediately afterwards, there is the reproduction process [89]. Usually after the cell replication, the host cell dies, freeing new microorganisms on the surrounding environment by keeping on infecting new close host cells and starting a new life-cycle [12]. ACE2 is a new important receptor for SARS-CoV-2, the same receptor of the SARS-CoVs [90]. The ACE2 receptor is on the mucosa of almost all organs: mouth, nose, throat, lungs, small intestine, colon, thymus, bone marrow, spleen, liver, kidney, brain, and heart (especially on the endothelium of the coronary walls and smooth muscle cells of the vessels wall) [90,91]. ACE2 receptors operate in the renin–angiotensin system (RAS). Renin is an enzyme regulating the blood pressure and encouraging the transformation of the angiotensinogen in angiotensin I. The enzyme ACE2 (a dipeptidyl carboxypeptidase) breaks the connection between proline and the residual carboxy-terminal of the phenylamine of the angiotensin II (eight amino acids), and it is transformed in angiotensin1-7 (seven amino acids). The ACE, in turn, divides the connection between phenylamine and histidine of the angiotensin I (10 amino acids) by transforming it in angiotensin II (eight amino acids). This induces an increase of blood pressure, by acting on the walls of the endothelium smooth muscle [90]. It is ascertained that the enzyme ACE2, an activator of the angiotensin II in angiotensin 1–7, represents an access site for the virus belonging to the coronavirus family, SARS-CoV-2. Some study of Kuba et al. performed on SARS-CoV and MERS-CoV showed that the ACE2 receptor shows a down-regulation after the bond with the virus [13] The ACE2 receptor “*attached*” by the virus goes inwards by reducing its ability to work. This may lead to less transformation of the angiotensin II in angiotensin 1–7. The angiotensin II links to the AT1 receptors, and this causes vasoconstriction, fibrosis, and pulmonary oedema, so that the down regulation of the ACE2 would reduce the synthesis of the angiotensin 1–7, which has some opposite effects: vasodilatation, antifibrotic, and antiproliferative. In such dynamics, the administration of the sartans, by blocking the pulmonary AT1 receptors, would lead to a reduction of the inflammation and the lung lesions as shown in Figure 1 [10,90,91]. The angiotensin II type-I receptor blockers (sartans) have been proposed to be a potential drug for the control of the angiotensin II serum levels that have been correlated with SARS-CoV-2 viral load and the severity of pulmonary correlated issues [92]. The protective effect of sartans in hypertensive patients affected by COVID-19 has been reported in clinical studies; it has been correlated to a positive action on the ACE2 receptors that represent the main route of infection of the SARS-CoV-2 virus [93].

**Figure 2:**
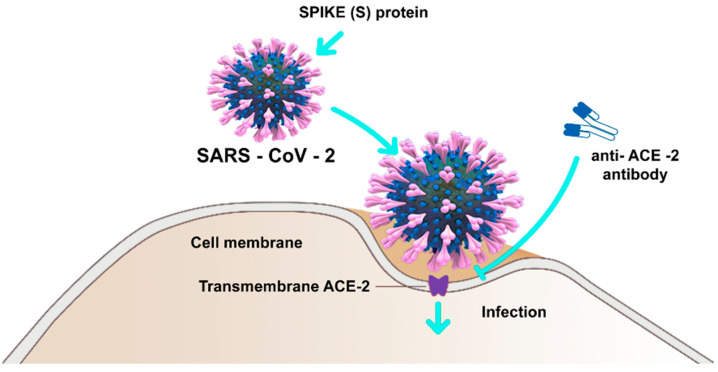
Mechanism of bond spike-cell receptor Ace2, figure designed by Giovanna Dipalma.

**Figure 3:**
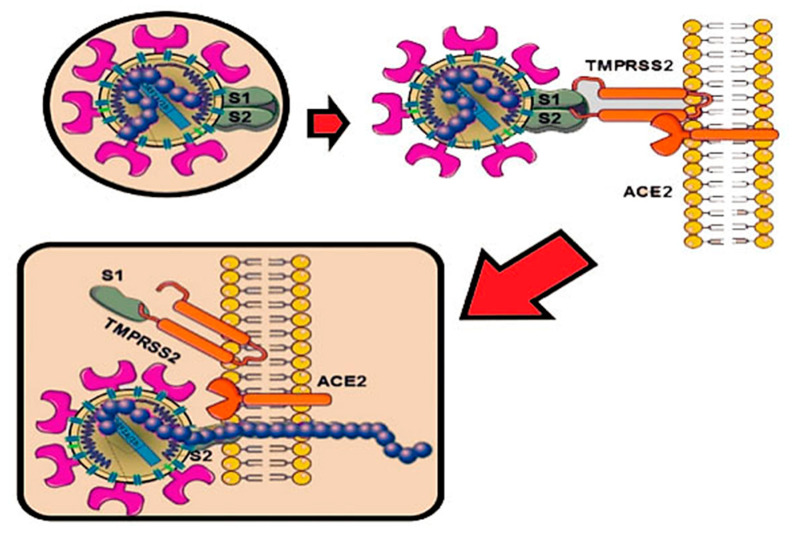
Mechanisms of access of SARS-CoV-2 in cells through the receptor ACE2 cleavage of the subunit S1 and S2 though the protease TMPRSS2 AND FURIN and fusion with the cell membrane and access, figure designed by Giovanna Dipalma.

Therefore, the virus SARS-CoV-2, through the protein S, reduces the expression of the ACE2 enzyme by also mitigating the potential as well as the number of available receptors, with a subsequent increasing of the angiotensin II and worsening of the lung lesions [91]. A particular attention has been given to the proteins S or spike placed on the envelope and the bond with the receptor (angiotensin-converting enzyme 2) ACE2 of the host cell, ensuring the connection and the invasion of the virus in the cell. It was already observed since the previous SARS-CoVs that the proteins S of the virus were related to the receptors ACE2, mainly in the lungs and intestine. It has also been shown that there is a significant presence of the oral mucosa, by highlighting it as an important site in the spreading and developing of the SARS-CoV-2 infection [94]. The viral protein S (spike protein), placed on the envelope, binds to the ACE2 receptor of the host cell. The protein S of the SARS-CoV-2 virus is structurally similar for 76.5% to the one of SARS-CoVs and MERS-CoV [15,90]. The authors have noted that the energy released by the hydrogen bonds with the protein S of the virus SARS-CoVs on the human ACE2 (receptor for human infection) is of −50.6 kcal/mol, which represents a significant value of affinity of the bond with human ACE2, but weaker than the proteins spikes of SARS-CoV-2 with ACE2 virus (−78 kcal/mol). This leads to confirming one of the higher affinities of the proteins of spikes of the SAR-CoV-2 virus than the Sars-CoVs [91]. Thanks to the techniques of cryogenic electron microscopy, so-called spike surface markers have been detected, distributed on the virus surface as a corona (causing the name of the virus) [95]. As the invasive nature of the host cell plays a fundamental role for the infection, it has been stated that “*spikes are the main target of the neutralized antibodies*, *so they are important for the study of vaccines and therapies*” [96]. Scientists observed a peculiarity of SARS-CoV-2, not noticeable in other similar coronaviruses, such as the SARS-CoVs, but in other influenza non-SARS viruses, which is the presence of a cleavage site similar to the furin in the glycoprotein spike (PS), between the two subunits S1 and S2 of the 2019-nCoV [97]. Furin is an enzyme in the cells, belonging to the class of hydrolase that catalyzes the release of ripe proteins from the inactive precursors through the break of the bond Arg–Xaa–Yaa–Arg (Xaa maybe any amino acid and Yaa arginine or lysine) [97,98,99,100]. The furin is an enzyme included in lots of human tissues; it would maybe make the virus able to infect several organs and give a high ability of transmission [99,100,101,102,103,104,105,106], as it has been observed that it may be possible also to find it in other viruses that infect with a relative easily, among those the influenza ones (in this case they are not placed on the spicules, but on a protein called hemagglutinin) [104,107]. The regulation of the viral cells by the protease is a control process, which is usual among the viruses [106]. It is often necessary for the maturation and infectivity of the virus. An important group of proteases of the host cells, used by several viruses, is the family of proprotein convertase (PC), including furin, PC4, PC5, PACE4, and PC7. It has been shown that when PCs develop viral proteins, some viruses become more infective and pathogenic. The majority of studies made on the maturation of virus by the PCs has been focused on the furin [104]. Several families of virus use the PC of host cells to manage their access process of the cells [106]. The enzymatic cleavage of the peptide bond by the PC encourages the bond and the fusion of the viral particles with the target cells [106]. According to another study of Coutard et al., issued on Antiviral Research, the presence of the cutting site for the furin may influence the lifecycle and the pathogenicity of the coronavirus [100]. Practically, the furin has a key role in the viral infection by cutting the glycoproteins of the viral envelope and improving the infection with the host cells [107]. The cleavage operated by the furin allows a transmissibility and invasion 1000 times more than the SARS-CoVs. Moreover, the furin determines the systemic diffusion of the virus, as the protease is involved in the activation of many functional activities [104]. The furin is considerably present in the lung tissue, in the intestine, and the liver, this would make those organs as potential target of the 2019-nCoV infection [97,108]. The access of the virus through the ACE2 receptors shall be encouraged by the presence of some protease placed on the cell surface, in proximity, almost attached to the ACE2 receptor (Figure 2). Among those proteases, in particular, we can find the serin-protease TMPRSS2 (protease transmembrane, serine 2) acting in synchrony with the ACE2 receptor and the furin [109,110,111] (Figure 3).

**Figure 4:**
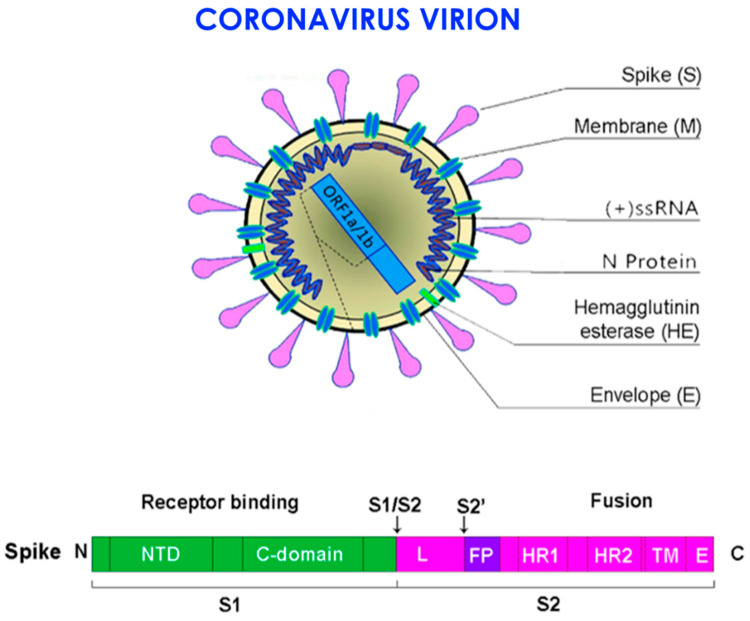
Scheme of the structure of the coronavirus and the glycoprotein spike of the coronavirus (S). The protein S is placed inwards on the envelope in order to make a corona structure. The protein S is made by two subunits, the subunit bonding the receptor S1 and the subunit of fusion S2. The protein hemagglutinin esterase (HE) is only present in the lineage A betacoronavirus. Figure designed by Giovanna Dipalma.

**Figure 5:**
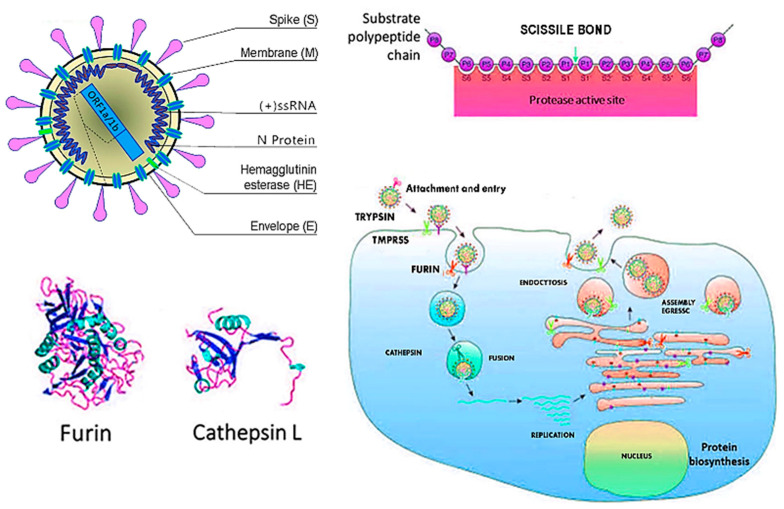
Scheme of the structure of the glycoprotein spike of the coronavirus (S) and protease of the host cells involved in the activation of the protein spike coronavirus (S). The protein S is placed outwards on the envelope to make a corona structure. The hemagglutinin esterase (HE) is only present in the lineage A betacoronavirus. The hemagglutinin esterase (HE) is only present in the lineage A betacoronavirus. The protein S is made by two subunits of fusion S2. Scheme of three protease of host cells noted activating the bond and fusion of the coronavirus s: trypsin, furin, TMPRSS, and pro-forma of *cathepsin* L. Figure designed by Giovanna Dipalma.

For the production of antiviral drugs, it is important to understand the particularity of penetration and fusion of the virus in the first step. A study performed by Madu et al. about the MERS-CoV infectivity has pointed out the mutation of two structural aminoacids of the protein S (G766R e L981P) [112]. The mutation G766R is found in the bond site with the furin by influencing its sensitivity and encouraging the cell access [109,113]. The use of cathepsin L endosome in the cell invasion is completely changed [114]. Park et al. have reported that if the protein S is divided by the furin during the first cell access step, the protease of the cell surface encouraged this access, and the MERS enters the cell faster. Those studies showed that the infectivity of the virus depended on the expression of TMPRSS, by keeping the cleavage site and Furin [112,115,116]. The enzymatic action of TMPRSS2 is fundamental, as SARS-CoV-2 infects the lung cells. SARS-CoV-2 may use the TMPRSS2 for trigging the S protein [111]. A study highlighted that the TMPRSS2 is an important element of the host cell, as it is essential for spreading a great number of viruses causing potentially significant infections, as the influenza virus A and coronavirus [114]. There are important data showing that the TMPRSS2 is not involved in the development and homeostasis, and so it is a potential and sensitive pharmacological target that may activate the first infective step of the circuit, namely the entry of the virus in the cell. In this regard, it is important to underline that the inhibitor of the protease serin, camostat mesilate, blocks the activity of the TMPRSS2 [114]. This drug (or similar), with probable antiviral activity increased, may be used for the treatment of patients with infection from SARS-CoV-2 [114]. A study reported that the activation mediated by the furin of the site S1/S2 in the infected cells may activate the subsequent entry, depending on the activation of the TMPRSS2 in the target cells [114]. The proteins S are made by two subunits, S1 and S2. The S1 links the receptor-binding domain (RDB) of the ACE2 on the target cell [91]. Together with the bond of the spikes with ACE2, the serin protease TMPRSS2 and the furin determine a change of the structure of the S2, allowing the fusion with the membrane of the host cell, allowing the access of the transfer of the viral content [105,117] (Figure 4 and Figure 5).

Furin expression has been found by the epithelium of the human tongue and in significant quantities in the squamous cells carcinoma (SCC) [94,102,118,119,120,121,122]. The tongue is at high risk of infection from coronavirus, and the presence of the SSC also increases the risk once exposed to the coronavirus [118]. There is a cleavage site on the spike that is similar to the furin, allowing the cleavage of the subunits S1 and S2, and it allows a high spreading of the 2019-nCoV virus [119]. It is not assured that this is the factor allowing a high spreading and infectivity of the virus [123]. It would be noted in a study in which this site would be eliminated, assessing the effects, and by blocking the furin and interacting with and pointing out its action mechanisms, it would allow the production of drugs and vaccines that are effective against the disease COVID-19. Probably, the evaluation of the three dimensions model of the structure of the protein spike could be important in finding solutions and potential remedies to neutralize the coronavirus. In a 2011 study about coronavirus infections on macaques affected by severe lung infections, some researchers tried to understand the reason why the saliva droplets were a source of contagion [33]. It has been found that epithelial cells of the salivary glands covering the conduits of the salivary glands had a high expression of ACE2 by resulting from the first target cells in order to become probably the first productive source [33,34,123]. The expression of ACE2 in human organs has been analyzed by considering the assumed data from the portal Genotype-Tissue Expression (GTEx) [124]. The ACE2 expression in minor salivary glands was higher than that found in lungs. This suggests that the salivary glands may be a potential target for SARS-CoV-2. Moreover, the RNA SARS-CoV-2 may be found in the saliva before lung lesions appear [123]. This may explain the presence of asymptomatic infections [125]. It is possible to suppose that for SARS-CoV-2, the salivary gland may be not only the first access site, but also one of the main and starting reproduction sources of growth, which would make saliva highly infective and infecting [33,126]. The high presence of the coronavirus SARS-CoV-2 in the saliva of the patients may even reach 91.7%, and from saliva, the virus is cultivated in vivo too [34]. This suggests that the SARS-CoV-2 transmitted by asymptomatic patients may come from infected saliva. Indeed, the greatest means of transmission of the virus occurs through micro droplets (Flügge droplets) and/or aerosol [2,33,127,128]. Cough, sneezing, and saliva are the main means of transmission [2]. The size of the droplets, the speed of sedimentation, and the rate of moisture of air determine the distance and duration in which the particles stay suspended in the air. With the diameter > 5 µm, the droplets may spread up to 1 m [39,127]. The droplets with a diameter of >5 µm (aerosol) have been characterized by a capacity of greater diffusion that could overcome more than 3 m of diffusion range (Figure 4). Then hands, by touching contaminated surfaces from the virus, transmit the virus to the mucosa (mouth, nose, and eyes) [39,47,127,128]. In a lowest percentage, the contact occurs through the fecal–oral route. The propagation of the SARS-CoV-2 virus through the droplets may occur through patients with or without symptoms, also showing the same period of incubation [129]. The infection through the droplets may occur by direct contact or for a distance lower than 2 m for an exposure higher than 15 min or for direct contact with infected hands. Even the use of air conditioner may be the cause of the diffusion of the virus, by aspiring the infected particles in the air and by strongly rejecting them in the environment at a distance of 8 m, so it becomes potentially more infective than the 2–3 m of a sneeze [39,47]. The time of incubation varies from five to 14 days both in symptomatic and non-symptomatic patients [129]. The identification of the virus of the epithelial cells of the lung may be found after 96 h since the contagion, while a lower time in oral mucosa and in conduits of the minor salivary glands, while 24–48 h after the showing of symptoms [129]. The first most frequent symptoms are fever > 37.4 °C, dry cough, dyspnea, asthenia, diarrhea, muscle pain, vomit, ageusia, and anosmia [60]. Therefore, the cause of the asymptomatic infection may come from the salivary glands. We should not ignore the infectivity of the single saliva [33,123].

## 3. The Cytokine Storm Syndrome (Css)

The cytokine storm leads to the interleukin release (IL)-6, IL-1, IL-12, and IL-18, together with the tumour necrosis factor alpha (TNF-α) and other inflammatory mediators [130,131,132]. The increasing lung inflammatory response may cause an increasing alveolar–capillary gas exchange, making hard the patients’ oxygenation of severe patients. There is a collapse of the lung walls and a severe bilateral respiratory insufficiency, and lesions to many organs with severe functional deficits [133,134,135]. Severe lymphopenia and eosinopenia [136] cause a decay in antiviral immunity and immunity in general. The recommendation is early screening for inflammatory markers, ferritin, c-reactive protein (CRP), and D-dimer 1 [137]. Helper cells of type 1 mediate the delayed inflammatory response, causing the IL-6 activation and other pro-inflammatory cytokines. If it is not treated, the inflammatory reaction may lead to severe lung lesions [138]. The insignificant increase of serum markers before starting the treatment with hydroxychloroquine and azithromycin may lead to deleterious adverse effects; moreover, it may be appropriate to make a differential diagnosis with the active tuberculosis and active fungal infections [139,140,141,142,143,144,145]. The use of IL-10 instead tends to conclude the blocking process of IL-6 by tocilizumab and avoids the formation of damaged interstitial lung tissues in fibrotic tissues. IL-10 is among the last potential biological therapeutic agents [146]. In addition to its ability to regulate the functions of lymphoid and myeloid cells, IL-10 has a powerful anti-inflammatory activity both in vitro and in vivo [146]. In this context, IL-10 has been identified as a potential therapy for inflammatory diseases involving type T helper 1 (Th1) and macrophage responses. In addition, the severity of the onset of secondary bacterial pneumonia during or shortly after COVID-19 infection is determined by a complex interaction between virus, bacteria, and host [147]. The host remains more susceptible to bacterial infections for several weeks after eliminating the virus, which indicates that increased susceptibility is not only due to an increase in viral virulence; in fact, it is known that the infection increases adherence and subsequent colonization with bacterial respiratory pathogens. Bacteria can adhere to the basement membrane after disruption of the epithelial airway layer due to the cytopathic effect of the virus. It has also been suggested that the increased adherence is due to the upregulation of the receptors involved in the attack of these bacteria [148]. Alternatively, COVID-19 alters the host’s innate immune response to subsequent bacterial challenges by becoming more sensitive to bacterial components, such as staphylococcal enterotoxin B and LPS. Cytokines such as IFN-γ, TNF-α, and IL-6 are synergistically up-regulated by staphylococcal enterotoxin B or LPS during influenza infections. These data clearly indicate that COVID-19 significantly alters the innate immune response to bacterial infections in a singular and atypical way; to date, little is known about the mechanism by which the virus modulates the innate and acquired immune response to bacterial infections of the lungs [148,149,150,151,152,153,154,155,156,157]. In vivo, a large part of the destruction of tissues derives from an excessive and unregulated inflammatory response, mainly of a neutrophilic nature which, if not contained, generates tissue damage by lowering the protective and regenerative dynamics [158,159,160]. In addition, the airway epithelial cells of healthy individuals produce IL-10; however, the epithelial cells of COVID-19 patients are deficient in the production of IL-10. Extremely confirmed data from analogous studies with COVID-19 patients reported that the under-expression of IL-10 is mainly due to clones of T lymphocytes [124,154,161,162,163]. It has been shown that SARS-CoV-2 may infect the lymphocytes, therefore playing a role in the modulation of the autophagy. The use of the medicine targets to the autophagy represents an emerging topic [135]. During the acute respiratory distress syndrome (ARDS), the active lung epithelial cells, together with the adaptive immune and innate filtering cells, are the cause of the aberrant production of proinflammatory molecules (cytokine storm), by encouraging an excessive recruitment of inflammatory cells and the local release of protease and oxidants, which are involved in lung manifestations of the disease [152,164]. With these premises, several cytokines, including IL-6, TNF-α, and IL-1β, are the cause of the inflammatory events associated to the disease caused by SARS-CoV-2 [165]. The local or systemic release of cytokines represents the most severe step of COVID-19. This process compromises the immune response against the virus SARS-CoV-2, giving rise to severe damage to the attacked organs, which leads to the death of the patient [166]. However, innate immune cells are populations that lead to production of cytokines, which respond to the inflammation and infection caused to the organs affected by the SARS-CoV-2, including the endothelial cells, the adipocytes, and mast cells. During the SARS-CoV-2 infection stage, the adipocytes produce IL-6, TNF-α, and IL-1β, by contributing to the worsening of the response of the host to the pathogens [167]. The autophagy was poorly considered and explored in COVID-19, while instead it is very involved both in the activation of lymphocyte and in the access and replication of the SARS-CoV-2 cells; therefore, the autophagy of lymphocytes plays an important role in the COVID-19. Therefore, the anti-rheumatic drugs, now recommended, are able to influence several biological processes, which intervene in the modulation of the autophagy in the lymphocytes and stimulate a reduction of the inflammation in patients infected by SARS-CoV-2 [135]. In healthy individuals, IL-10 has been shown to exert an inhibitory activity for the production of TNF-α, IL-1β, IL-6, and IL-8; therefore, it is possible that the constitutive production of IL-10, as occurs in the lungs of healthy people, may constitute an essential moment of homeostatic and anti-inflammatory balance [168]. In fact, experiments on IL-10 deficient knockout mice spontaneously develop inflammatory syndromes such as irritable bowel syndrome (IBS). Furthermore, in the lung context, IL-10 has been shown to be expressed by alveolar macrophages and stimulated by the bacterial lipopolysaccharide (LPS), by TNF-α [133,148,154,162,169,170,171,172,173,174]. As in the SARS-CoVs, even the 2019-nCoV may be transmitted in a quick way among human beings [34,175].

## 4. Therapeutic Approaches

From the beginning of the pandemic of COVID-19, a disruptive flow of diagnosis choices and therapeutic solutions after the several studies developed on the several clinical experiences arose during the two waves as well as the previous infections: SARS-Cov and MERS [176]. Currently specific pharmacological therapy for the treatment of the disease COVID-19 does not exist, although there are many pharmaceutical companies that are working hard on the vaccine production [25,177,178,179]. The purpose is mainly to provide a support therapy that can treat the symptoms to try to prevent respiratory failure [180,181]. The insulation of patients is essential to avoid transmission. The quarantine at home is compulsory to insulate asymptomatic and symptomatic people by associating a correct nutrition and hydration as well as symptomatologic therapy of cough, fever, and sore throat. Hospitalization is recommended in severe cases only [182,183]. The previous pandemics SARS-CoV or SARS-CoV and the in vitro observations have mainly provided the data for the pharmacological treatments for COVID-19, with use of antiviral, antinflammatory drugs, cell therapy, immunomodulators, and antioxidants [177,184,185]. The use of antibiotic therapy is recommended to avoid bacterial superinfection [186]. The use of steroids has to be assessed carefully, according to the case, by considering risks and benefits, as increases of mortality, secondary infections, disturbances of behavior, and hyperglycaemia occurred, by using a lower possible dosage and not for a long time [187,188]. The anticoagulant therapy is recommended in patients with COVID-19 in the early stage. The risk of ischemia and dissemination intravascular coagulation increases in case of infection, inflammation, and other factors related to the disease [189]. In addition to the intensive care, the use of transfusion of convalescent plasma (CP) is assessed to save severe patients. By using a dose of (200 mL) of CP on 10 severe patients, it has been observed that the dose was well tolerated, and the results have shown an increase and a significant maintaining of the neutralized antibodies by detecting the disappearance of the viremia in seven days. In the meantime, patients improved the clinical symptoms, and the clinical parameters quickly improved in three days. To the radiological exam, several degrees of absorption of the lung lesions were evident within seven days. These results indicate that the CP may be considered a promising choice of treatment for the severe COVID-19, even if some of them are now the object of study and experimentation [190]. Among the post-COVID-19 problems that are not well known, even by the doctors, and that have poor media coverage, there is another category of patients, called “*long-haulers*” [191]. This is a population of patients who, eight or nine months after the initial infection, which is often slight or moderate and so never hospitalized but treated at home, do not reach healing. These patients had suffered no damage in several organs by the “*cytokine storm*” of the COVID-19, as Alessandro Santin, who is responsible for the team of research of the Smilow Cancer Center and director of the department of oncology of Yale School of Medicine, states in his interview [191]. The majority shows dyspnoea (shortness of breath: SOB) by limiting the working capacities and any other kind of physical activity. There are some chronic symptoms such as constant chest and heart pain, intestinal disorders, headache, difficulty concentrating, memory loss, and tachycardia [192,193]. The virus would remain in short quantity in some organs of “long-haulers” (which are “*not*” usually infective), so that the immune system can detect its presence. The mast cells and macrophages (cells of the immune system) are led to produce not a “*cytokine storm*” but a “*rain of cytokine*”, a sign of persistent chronic inflammation that causes asthenia and difficulty healing [191,194]. They are patients who are treated with anxiolytics due to being wrongly diagnosed as anxious or hypochondriacs. In the case of immunosuppression, for the persistence of the virus, these patients may infect again. An important study by Larry Afrin, expert in mast cells, has detected that by using the antihistamines in these “*long-haulers*”, their life quality improves significantly [195]. It is assessed that more than 30% of patients infected in slight and/or moderate form may belong to this kind of patient.

## 5. Resveratrol Adjuvant Supplements for COVID-19

Resveratrol (trans-3,5,4′-triidrossistilbene) is a natural polyphenol, which is presented as a phytoalexin and is characterized by several properties. Resveratrol is present in several species of plants such as huzhang (polygonum cuspidatum), cranberry (vaccinium macrocarpon), red mulberry, especially present in the seeds and in the peel of red grapes (Vitis vinifera), and in red wine [196]. Resveratrol is poorly soluble in water and poorly bio-available in the oral cavity [197]. Resveratrol is quickly metabolized in the liver in glucuronides and sulphates; for this reason, it is considered toxic at high concentration. The type of feeding and the individual differences in the metabolism detected its big influence on the bioavailability [197]. In another study about resveratrol, 30 min after orally consuming red wine, the bioavailability of the resveratrol was only in few traces, while a few instants after, the glucuronides, metabolized by the liver, were abundantly in the circuit for a long time [197]. Recently, the use of structured nanoparticles is being shown to improve the bio-availability of resveratrol by extending the release in vivo. The nanoparticles of solid lipids (SLN) and carriers of nanostructured lipids (NLC) added to the resveratrol allowed the entrapment of 70% and a stability up to two months [197]. The studies in vitro showed a slow and prolonged release of resveratrol in the gastro-intestinal tract. The resveratrol shows good effects in inflammatory conditions (it reduces the production of nitric oxides), oncology (it reduces the release of free radicals), cardiovascular diseases (it protects the cell death induced by the reactive oxygen species ROS, by activating the AMP-activated protein kinase (AMPK) in the heart muscle cells (H9c2)), obesity, type 2 diabetes, and neurodegenerative diseases [198,199,200,201,202,203,204]. In particular, the capacity of the resveratrol to inhibit the growth of bacteria, fungi, and viruses has been widely studied. According to Lin and his team through studies in vitro, the resveratrol limits the infections caused by more pathogenic agents, such as helicobacter pylori, staphylococcus aureus, or toxoplasma gondii [199]. The RVS was shown to provide an antiviral effect against several infections, including the virus Epstein–Barr (EBV), the enterovirus 71 (EV71), and the herpes simplex virus (HSV), as well as the respiratory viral infections caused by influenza, respiratory syncytial virus (RSV) and rhinovirus, the MERS-CoV (Middle-East respiratory virus-coronavirus), the human metapneumovirus, and the severe acute respiratory virus coronavirus (SARS-CoV), and was also shown to reduce the inflammatory mediators released by the viral infection [205]. An in vitro study performed by Lin and his team about the influence of the resveratrol in the MERS-CoV infection may report valid supports for the therapeutic strategies for SARS-CoV-2. Those studies detected that the resveratrol inhibited in significant MERS-CoV infection. The cell death (apoptosis) caused by MERS-CoV in vitro significantly decreased after the virus infection [205]. Even the nucleocapsid protein (N) required for the replication of MERS-CoV was present in lower quantity after the treatment with resveratrol. The resveratrol at high concentration of 250 Mm has a lower toxicity (as it is noted that this substance increases the hepatic stress, because of the increased levels of hepatic enzymes) [206], but is negligible compared to the one caused by the MERS-CoV infection, which is much more important. Therefore, we may think that the treatment with resveratrol may be a good therapeutic practice. In a study, it was shown that the resveratrol may be administrated at high dosage up to 250 μM or at a relatively low concentration, as 62.5 μM, repeated more times, each 24 h, for the treatment of infected cells of MERS-CoV [198]. In possible antiviral mechanisms for the resveratrol, it has been reported that it activates the means of ERK1/2 indication, and it promotes the cell proliferation and improves the SIRT1 indication, both correlated to the cell survival and the repair of the genomic sequence in response to the damage to the chain [198]. On the other hand, the resveratrol may contrast the apoptosis induced by MERS-CoV by regulating in a reduced way the indication of FGF-2. Moreover, the MERS-CoV infection may lead to the production of inflammatory cytokines, while the resveratrol may reduce the inflammation by interfering with the via NF-KB. In those studies, the levels of enzyme cleavage of caspase 3 have been reduced by the resveratrol after the MERS-CoV infection [205,207]. This may be the result of the direct inhibition of the cleavage of the caspase 3 through the restoration of the cell surviving and so the decrease of apoptosis induced by the virus or a probable block important for the cleavage of the caspase 3. Actually, on the basis of the results of those researches, what it is interesting to see is how we may find as a mechanism even in the SARS-CoV-2 infection as well as the anti-Mers-CoV effect, a mechanism that may require further scientific contents [207]. Its main antiviral mechanisms are linked to the inhibition of the viral proteic synthesis and the inhibition of several ways of transcription and transmission of signals, by inhibiting the viral genic correlated expressions [207,208]. It has been shown that the resveratrol inhibits the cell growth during the steps G1 and G1/S, and it is an anti-inflammatory mediator through the inhibition of the activity of the nuclear factor NF-κB during the activity of the procyclooxygenase-2 and the production of prostaglandin [207,208]. After those mechanisms, the resveratrol determines a “down-regulation” of the apoptosis of the cell induced by MERS-CoV in vitro. Its capacity to experimentally disactivate the renin–angiotensin system (RAS) and increase the quantity of ACE2 [209] has been detected. It has been found that the therapy with the resveratrol significantly decreases the levels of angiotensin, renin, ACE1, and AT1R, and it increases the levels of ACE2, AT2R, and MAS1 [205,210]. By virtue of those results, it would be appropriate to also verify the capacity of the resveratrol on the SARS-CoV-2 virus. As polyphenols have anti-inflammatory, immune-stimulating, and antioxidant effects, we are moving towards the evaluation of the effects on severe lung caused by SARS-CoV-2 [209]. As there are some studies about the anti-inflammatory and repairing effects of the resveratrol on the oral cavity tissues (112), by correlating the high rate or receptors ACE2, the acclaimed targets of SARS-CoV-2, and its capacity of the resveratrol to increase the quantity of ACE2, we may assess the reduction of the link of the coronavirus SARS-CoV-2 on the oral mucosa sites by the resveratrol [196,208,210,211,212].

## 6. Vitamin D Supplement for COVID-19

There are interesting studies on the action of vitamin D in the COVID-19 infection. Some studies reported that vitamin D may be used to decrease the acute lung damage induced by the lipopolysaccharides through the renin-angiotensin mechanism. The calcitriol (1,25-dihydroxyvitamin d3) exercises effects on the axis ACE2/ANG (1–7)/MasR [213,214,215]. The vitamin D improves the ACE2 expression and is involved in the immune system, as it modulates the response of the body to an infection. COVID-19 is not only due to the virulence of the pathogenic agent, but also to the release of pro-inflammatory cytokines. Some studies have described that the lack of vitamin D alters the capacity of maturation of macrophages and alters the capacity to originate antigens of surface with specificity for the macrophages, produce phosphatase lysosomal, and produce hydrogen peroxide (further antimicrobial function) [214,215]. In these studies about the vitamin D, it has been noted that the most vulnerable population group for the COVID-19 is the oldest population and also presents the lowest levels of vitamin D (the lowest levels of vitamin D in elderly are seen in Spain, Italy, and Switzerland) [213]. Moreover, the vitamin D has already been shown to be able to protect from the acute respiratory infections, and it does not damage the body [213,215]. Therefore, the vitamin D may control the fusion mechanism of SARS-CoV-2 through the inhibition of the ACE2. Therefore, some substances that block the receptor of the angiotensin may be used as promising therapies against SARS-CoV-2 [215]. However, several issues about the wide heterogeneity of the dietary supplements and administration doses could represent a strong limit for the generalizability of their anti-inflammatory, immune-stimulating effects on COVID-19 subjects.

## 7. Melatonin Supplement for COVID-19

Melatonin (N-acetyl-5-methoxytryptamine) is a lipo-water-soluble hormone secreted for a wide part by a small gland in the brain called the pineal gland or epiphysis. The melatonin production is low in the first months of life, increases at a young age, and decreases in older age [216]. Melatonin is characterized by anti-inflammatory and antioxidant properties through several mechanisms [217,218]. This hormone stimulates the increase and maturation of NK cells, T and B lymphocytes, monocytes, and granulocytes in the bone marrow and in other tissues [219]. In literature, it has been reported that the administration of melatonin also revealed an increase in the number of macrophages/monocytes and an upregulation of the antigenic receptors of the mono-cell/macrophage line [220]. Furthermore, inflammation is commonly associated with a high production of cytokines and chemokines, while melatonin causes a reduction in proinflammatory cytokines TNF-α, IL-1β, IL-6, and IL-8 and an increase in level of anti-inflammatory cytokine IL-10. In fact, the melatonin reduces serum levels of highly sensitive C-reactive protein and suppresses nuclear factor NF-κB [215,217,218]. The melatonin administration has been associated to a reduction of the risk of ARDS (acute respiratory distress syndrome) and therefore also mortality; the melatonin also reduces the risk of haemorrhagic shock during viral infections [221,222]. The melatonin also acts by inhibition of calmodulin in association of an increased presence of ACE2 by enhancing its binding on the cell surface, which represents the host cell receptor for SARS-CoV-2 [223]. The melatonin may also block CD147, another cellular receptor for SARS-CoV-2 involved in the regulation of chemotaxis and lung inflammation [224].

In lung diseases, the presence of ACE2 and CD147 receptors determine vascular permeability and cause pulmonary oedema. These receptors intervene by activating the renin–angiotensin–aldosterone system (RAAS) and contribute to the appearance of severe lung damage [225].Furthermore, in vitro studies reported that melatonin inhibits the SARS-CoV-2 major protease (Mpro). The Mpro is an ever present enzyme among the coronavirus species; thus, melatonin identifying itself as an inhibitor of Mpro would become a broad spectrum drug of SARS-CoV-2 [226]. Moreover, the bats, the main reservoirs of coronavirus, have almost no symptoms associated to the viral infection [227]. In mankind, newborns have higher melatonin levels than the adult population, probably contributing to the clinical manifestation of more marked symptoms in the latter age group [228,229]. Furthermore, exogenous administration of melatonin has been shown to increase the production of antibodies [230], both in physiological and pathophysiological conditions. Melatonin has immunomodulatory effects. In summary, melatonin exerts an immunostimulant effect in cases where the immune system is suppressed, and is immunosuppressive in cases of in-inflammation [231]. In addition to the anti-inflammatory and pro-inflammatory effects, melatonin has antidepressant, anxiolytic, neuroprotective, and antihypertensive properties, which certainly improve the clinical performance of the sick patient [232]. Due to its various characteristics, therefore, it has been estimated that melatonin can be used as a prophylaxis or treatment in COVID-19.

## 8. Lianhuaqingwen (LHQW) Herbal Compound

Traditional Chinese medicine (TCM) is playing an important role in the treatment of COVID-19, for the prevention and control of the spread of COVID-19 [233]. The lianhuaqingwen (LHQW) is a product of TCM, and the LHQW capsules contain a mix of plant extracts whose active ingredients refer to polyphenols, triterpenes, anthraquinones, iridodiades, more than 12 types of plants, and 61 active ingredients with beneficial effects. The commercial production of the LHQW capsules is packaged according to the Chinese Pharmacopeia [234]. The National Health Commission has approved LHQW for the treatment of COVID-19 [235], and in vitro and human studies reported the effectiveness of this product against COVID-19 [235]. Clinical studies have shown that LHQW is able to reduce the cytokine release that is associated to the lung damage related to infiltration and increase in inflammatory cells [176]. The LHQW can be administered not only in patients with mild clinical symptoms such as fever, fatigue, and cough, but also for severe patients with lung lesions. Clinical studies have shown that the LHQW associated with conventional Western medicine in the treatment of pneumonia improved the results of clinical treatment (91.5% vs. 82.4%); reduced the median time of symptoms. (seven vs. 10 days); shortened the time of fever (two vs. three days), cough (seven vs. 10 days), and fatigue (three vs. six days); and lead to the disappearance of rattle and wheezing. Moreover, an improvement of the tomographic evidence (83.8% vs. 64.1%) was reported, and the CRP index improves with consequent recovery of patients with pneumonia with a faster clinical recovery (78.9% vs. 66.2%). No serious adverse events were reported [235,236]. In light of safety and efficacy evaluations, the LHQW administration could be considered useful for the treatment of COVID-19 to improve the clinical symptoms [235]. Recently, the gamma-oryzanol has also been hypothesized by traditional Chinese medicine as therapeutic for cytokine storms in COVID-19 according to its anti-inflammatory, antioxidant activities and neuroprotective functions [237,238].

## 9. Novel Orientation for Infection Prevention

As it has been noted that the lower human respiratory tract is protected by the IgG (IgG1 is the most spread), while the upper respiratory tract (mucosa of the oral cavity and nasal airways) is protected by secretory IgA1 (sIgA1), researchers have thought of a scientific solution that may block the entry of the virus through the ACE2 receptors and the lipid raft, with a specific substance that may destroy the virus SARS-CoV-2 in the first access ways [239]. The virus SARS-CoV-2 is able to initially attack the cells of the epithelium of the airways by tying its Spike protein (S) to the angiotensin trans-membrane I by converting the enzyme 2 (ACE2) with the intervention of the protease of the serum transmembrane 2 (TMPRSS2). The two proteins, ACE2 and TMPRSS2, are placed in the lipid raft, rich in cholesterol, of the cell membrane [111,240].

The two main processes which influence the pathogenesis SARS-CoV-2 are:

(1) The entry in the cells through endocytosis.
(2) The trigger of an exaggerated inflammatory response [241].

A spray has been developed by a research group directed by the Dr. Matteo Bertelli and Prof. Tommaso Beccari of the University of Perugia, called “*Endovir Stop*”, containing composites inhibiting both the endocytosis and the inflammatory response, which is responsible for the oxidating damage as preventive measure for the infection of SARS-CoV-2 and contributing to reducing the viral load of the mucosa of the oral and nasal cavity [242].

The spray compound has been approved by the Italian Ministry of Health on 28 May 2020, with the number 0019821 as dietary supplement [243], as the two basic substances of the spray are:

(1) Alpha-Cyclodextrin (mg 1.5)
(2) Hydroxytyrosol (mg 5).

The α-cyclodextrin is approved for its peculiarity to exhaust the sphingolipids, which together with the cholesterol form the lipidic rafts where the ACE2 is placed [243]. Additionally, in the formulationm the hydroxytyrosol has been included, a substance extracted by the leaves of olive and fruit. This has anti-inflammatory and antioxidant properties (active compounds: 25% hydroxytyrosol 5 mg, α-cyclodextrin 1.5 mg, water; sweetener: fructose; co-emulsifier: glycerin; aromatization: lemon flavor, acidifier: citric acid; preservatives: sodium benzoate, potassium sorbate; control of viscosity: xanthan; sweetener: glycosides steviol, sucralose) [244,245]. From the study group of Perugia of Professor Bertelli, it has been observed that Endovir Stop is safe and does not have cytotoxic effects, so it promises a good efficacy, also because it has been seen in vitro that the virus in contact with these two substances immediately dies [244,245]. Endovir Stop has been produced by the Ebtna-Lab of the Group Magi of Bolzano, yet committed since then to the study of viral endocytosis through the lipidic rafts, areas of the cell membrane particularly rich in cholesterol and proteins [242,244]. Because of the pandemic of COVID-19, researchers, by studying the virus, have underlined the modality with which the coronavirus enters our cells. They have noted that not only the ACE2 receptor, but also the lipidic rafts, are access of SARS-CoV-2 [244,245,246,247,248,249]. The study of the Dr. Bertelli has shown that these natural molecules “*impede the entry of the SARS-CoV-2 virus according to the process of the endocytosis lipid-raft mediate*” [244]. Therefore, as well as the already confirmed and main measures of prevention, distancing, masks, sanitizer gel, and vaccines, a valid help may come from a new service, Endovir Stop, which uses the polyphenols of the olive oil of Garda and cyclodextrins [244]. Unfortunately, the WHO has recognized another phenomenon, “pandemic fatigue”; people feel demotivated in following behavior recommended to protect themselves and other people from the virus, because they have been subjected to limitations for more than eight months. Therefore, some strategies have been created to ensure the public support for the preventive measures for COVID-19 [250]. An important consideration to make is about the control. Countries that have maintained low per capita mortality rate of COVID-19 seem to have shared some strategies that require immediate surveillance, test, tracking of contacts, and strict quarantine. The adoption of the digital technology and its placement in the politics and health system effectively upgrades the data management and the degree of coordination, planning, and strategies of surveillance. Artificial intelligence may improve the quick diagnosis and the prediction of the risk of COVID-19 together with the strict collaboration of all the citizens in maintaining a right behavior [251].

## 10. Conclusions

The enzymatic inhibitors are the first to be studied as probable therapy against SARS-CoV-2 as drugs interfering in the mechanism of interaction ACE2, furin, and TMRPPS2, requiring the control of several pharmacological properties, paths of drugs transportation, blood circulation of drugs, metabolism of drugs, and side effects coming from the drugs’ interactions with large variety of enzymes. Therefore, some thermodynamic and kinetic data in the building of the drug during the preclinical studies are necessary. Novel adjuvant therapies are currently investigated to improve the clinical prognosis of COVID-19 long-haulers and their chronic symptoms. Further randomized long term clinical trials are necessary to validate the adjuvant supplements against this disease.

## Data Availability

All experimental data to support the findings of this study are available contacting the corresponding author upon request.
